# RB in glutamine metabolism

**DOI:** 10.18632/oncoscience.39

**Published:** 2014-05-08

**Authors:** Brian Clem

**Affiliations:** Department of Biochemistry and Molecular Biology, James Graham Brown Cancer Center, University of Louisville, Louisville, Kentucky

In a majority of cancer cells, inactivation of the retinoblastoma protein (pRB) through hyperphosphorylation, deletion, or mutation leads to dissociation from E2F effector proteins and aberrant activation of downstream target genes. Suppression of pRB function ultimately results in the loss of regulatory control over multiple biochemical pathways required for tumorigenesis, including cell cycle control, apoptosis, angiogenesis, and metastasis. Notably, recent studies from numerous groups now are highlighting a functional role for the RB pathway in controlling not only neoplastic immortalization and transformation but also cellular metabolism.

Tumor cells predominantly exhibit a metabolic switch towards aerobic glycolysis (the Warburg effect), which is characterized by an increase in glucose catabolism to lactate at the expense of mitochondrial oxidation. While several oncogenes, such as *MYC* and *KRAS*, have been reported to mediate glycolytic flux to lactate, Hsieh *et al*. recently demonstrated that loss of pRB function resulted in inhibition of glucose oxidation within the tricarboxylic acid (TCA) cycle. Specifically, pyruvate dehydrogenase kinase (PDK4) was revealed to be a direct pRB-E2F-1 target gene and, when activated, suppressed PDH activity[1]. Importantly, as a result of the Warburg effect in tumor cells, diminished glucose availability for the TCA precipitates a need for other anaplerotic carbon sources to sustain mitochondrial function and macromolecules generated from TCA intermediates. This may be of increased significance in neoplastic cells harboring a dysfunctional RB pathway since pRB suppression has been reported to cause an E2F-1-mediated increase in mitochondrial oxidative capacity via activation of a myriad of genes including those responsible for mitochondrial respiration[2].

Glutamine is the most abundant amino acid in human sera and, while non-essential, through glutaminolysis can provide a ready supply of carbon for TCA anaplerosis and other cellular pathways. For tumor cells, enhanced reliance on this cascade leads to glutamine dependency for cell growth and survival. Similar to glucose, glutamine catabolism is a regulated process, and the signaling mechanisms that control glutamine metabolism are under intense investigation. Recently, we found that immortalized mouse embryonic fibroblasts (MEF) lacking all three RB family members (TKO: pRB, p107, and p130) exhibited increased glutamine uptake compared to isogenic wild-type MEFs. In the TKO MEFs, this was mediated through increased expression of the glutamine transporter, ASCT2, and glutaminase 1 (GLS1)[3]. In addition, loss of the RB family led to increased incorporation of glutamine into the TCA metabolite aspartate, a phenomenon also observed in RBF1-repressed *Drosophila* larvae and in pRB suppressed tumor cells[4]. We postulated that this increase in glutamine anaplerosis, coupled with the expected increase in pRB-regulated mitochondrial respiratory genes, would support mitochondrial activity in the TKO MEFs. Indeed, they exhibited not only an increase in basal oxygen consumption but, unlike WT MEFs, were capable of selectively utilizing glutamine for mitochondrial function[3].

**Figure 1:**
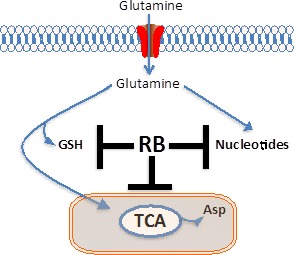
RB controls various pathways within glutamine metabolism Glutamine carbon is utilized in multiple biochemical processes including biosynthesis of glutathione, *de novo* nucleotide production, and for anaplerosis within the TCA. Neoplastic cells harboring inactivated pRB have an increased demand for nucleotide metabolism, which coupled with the requirement to maintain redox homeostasis and mitochondrial function ultimately leads to greater selective sensitivity to glutamine availability. GSH: glutathione, TCA: tricarboxylic acid cycle, Asp: Aspartate

Beyond the TCA cycle, glutamine also provides carbon for the *de novo* synthesis of macromolecules within other cellular pathways. Specifically, glutamine-derived glutamate is required for the production of glutathione, which is essential in maintaining redox homeostasis and facilitation of certain enzymatic reactions. Importantly, loss of RBF1/pRB expression either in *Drosophila* or mammalian cells increased glutamine incorporation into glutathione[3, 4]. In addition, glutamine withdrawal significantly reduced GSH levels within TKO MEFs and resulted in increased generation of reactive oxygen species (ROS)[3]. Together, these data suggest that in the absence of glutamine, RB-inactivated cells may be more susceptible to oxidative stress. While not directly examined in these cells, this is supported in that sensitivity of RBF1-depleted larvae, but not control animals, to fasting was rescued by supplementation of the anti-oxidant N-acetyl-cysteine (NAC). Notably, the survival effect of NAC was attributed to reduction of oxidative stress and suppression of GSH levels, thereby increasing the availability of glutamine carbon for nucleotide production. Nicolay *et al*. now have demonstrated that pRB-depleted cells have an increased requirement for nucleotide metabolism and combined with the necessity to maintain redox balance results in greater sensitivity to glutamine availability than normal cells[4]. Taken together, these studies strongly suggest that cancer cells harboring inactivated pRB may be selectively affected by targeted anti-glutamine strategies, including several glutaminase inhibitors that are currently in pre-clinical development (*i.e*. BPTES, c968, and CB-839).

Lastly, extensive *in vitro* studies have characterized several different tumor types as glutamine dependent. While a majority of these systems have dysfunctional pRB and the studies described here clearly portray a significant role of the RB pathway in controlling glutamine metabolism, evidence also suggests that the alterations in glutaminolysis in human cancers are cell context dependent. This is specifically demonstrated in the variability of glutamine flux either to glutathione or aspartate in response to pRB suppression in various human cancer cells[4]. These differences likely are attributed to the cumulative contributions of oncogenic/tumor suppressor functions in defining the overall metabolic phenotype. For example, in addition to aerobic glycolysis, both *MYC* and *KRAS* have been described to directly modulate glutamine catabolism and render cells glutamine “addicted”[5, 6]. In yet another layer of complexity, these signaling pathways exhibit considerable cross-talk, wherein, pRB can modulate Ras activity and c-Myc and E2F cooperate to transcriptionally activate certain target genes[7]. In summary, more extensive examination is needed, specifically in primary human cancers, to dissect the critical signaling networks that regulate glutamine catabolism in order to identify those populations that may be most responsive to specific anti-metabolic therapies.
